# Augmentation of intramedullary nail in unstable intertrochanteric fractures with plate or cable

**DOI:** 10.3389/fsurg.2024.1293049

**Published:** 2024-02-15

**Authors:** Yanrui Zhao, Hanzhou Wang, Yang Liu, Lei Shan, Junlin Zhou

**Affiliations:** Department of Orthopedic Surgery, Beijing Chaoyang Hospital, Capital Medical University, Beijing, China

**Keywords:** unstable intertrochanteric femoral fractures, lateral femoral wall, intramedullary nail fixation, clinical outcome, Harris Hip Scoring system

## Abstract

**Backgrounds:**

This study aims to evaluate the clinical outcome of intramedullary nail supplemented by buttress plate or cable in the treatment of intertrochanteric fracture with broken lateral wall.

**Methods:**

From May 2015 to January 2022, patients with unstable intertrochanteric femoral fractures underwent intramedullary fixations strengthened with buttress plates or cables, which depended on the lateral femur wall fragment type. The clinical and radiographic results were compared between the two groups. The hip function was evaluated according to the Harris Hip Scoring (HHS) system.

**Results:**

Forty-one patients who sustained intertrochanteric fracture + broken lateral wall were enrolled. Of these, thirty-four received a minimum of twelve months of follow-ups. No statistically significant differences in baseline and operative data were proved between these groups (*p* > 0.05). Three patients were observed fat liquefaction after surgery (plate group: 2 cases, cable group: 1 case). All patients could sustain partial/full weight-bearing and no case underwent subsequent operation. The HHS of the last follow-up presented 83.6 ± 4.9 points in the plate group and 83.8 ± 3.7 points in the cable group.

**Conclusions:**

Intertrochanteric femoral fracture with broken lateral wall is an unstable injury type, the operative treatments of which have been challenging and controversial over the years. Augmentation of intramedullary nailing system using plate/cable contributes to reconstructing the lateral femur wall.

## Background

Intertrochanteric femoral fracture is among the most frequent injuries in the elderly. Symptoms such as tenderness, swelling and motion disabilities occur after a falling, seriously affecting living quality and life expectancy (1). Some patients also suffer concomitant diseases, including pressure sores, pneumonia and urinary infections due to prolonged recumbency. Unstable intertrochanteric fracture (AO/OTA 31 A2 and A3 fractures) presents a broken lateral femoral wall. It cannot to provide biomechanical stability, which goes against the interoperative reduction and leads to the failure of internal fixations (2). A prior study demonstrated that 22% of patients with broken lateral walls received re-operations due to unsatisfied primary surgeries (3).

For stable type, dynamic hip screw (DHS) is considered the optional method with reproducible results. While in unstable type, extramedullary fixations continuously acquire poor clinical outcomes and have a high incidence of postoperative complications, especially medialization, coxa varus and screws cut-out (4, 5). The intramedullary nail is the first choice for this challenging fracture, providing sufficient biomechanical stability and causing minimal soft-tissue destruction (6–8). Additionally, this device allows patients to start early rehabilitation training and complete weight-bearing exercises.

The intertrochanteric bone cortex is thought to be the critical factor in preventing femoral head collapse and achieving anatomic reduction and is also considered necessary for fracture healing. Some authors found that the broken lateral wall could be damaged during the intramedullary nail, and screws were inserted (9). Therefore, there are still some challenges for unstable intertrochanteric fractures, and previous studies have reported that auxiliary plate or cable fixation is an option in the reconstruction of the lateral wall (9–11). However, there are few reports comparing the operative effect of these additional fixations in the treatment of intertrochanteric fracture with the broken lateral wall.

Based on this, our retrospective comparative study evaluated the clinical outcomes of intramedullary nail strengthened with a buttress plate or cable for unstable intertrochanteric fracture.

## Patients and methods

### Cohort identification

With the approval of the institutional review committee, this retrospective study was performed on patients who received proximal femoral nailing and lateral wall reconstruction for unstable intertrochanteric femoral fracture in Beijing Chaoyang Hospital between May 2015 and January 2022.

Inclusion criteria were as follows: (1) over 18 years old; (2) unstable intertrochanteric fracture: AO/OTA 31A2.2, 31A2.3 and 31A3 intertrochanteric fracture (12); (3) treated with intramedullary nail and lateral wall reconstruction with the buttress plate or cable. Exclusion criteria included (1) history of operation at the surgical site; (2) previous history of tumor in the injured limb; (3) open fracture; (4) stable intertrochanteric fracture: AO/OTA 31A1 and 31A2.1 (12); (5) conservative treatments; (6) follow-up of less than a year.

Patient demographics (age and gender), injury mode, injury side and surgical details (operative time, fluoroscopy time, time to surgery and blood loss) were acquired from medical records. All cases were analyzed by AO/OTA classification using preoperative x-ray and 3D CT imaging. Two resident physicians performed the fracture type clarification, and a senior physician was invited to evaluate when divergence appeared.

We used the additional titanium cable fixation for the apparent displacement of the lateral wall segment. Comparatively, we chose the buttress plate fixation for the comminuted lateral wall.

### Surgical procedures

All patients were under epidural/general anesthesia and took a supine position for traction. The C-arm machine was used to confirm the closed reduction quality. A 3 cm longitudinal incision was made above the greater trochanter to separate the subcutaneous and muscular tissues and expose the apex of the femoral greater trochanter. A guide needle was inserted into the slightly medial part of the greater trochanter apex. The canal was reamed through the direction of the guide needle and the calibrated reamer measured the length of the pulp cavity. We used the InterTAN (TRIGEN INTERTAN, Smith & Nephew, Memphis, Tennessee) as the intramedullary fixation. The proximal interlocking screws and distal locking screws were inserted in sequence. After confirming that depth and position were satisfactory, the incision was closed layer by layer.

### Cable group

**Figure 1:**
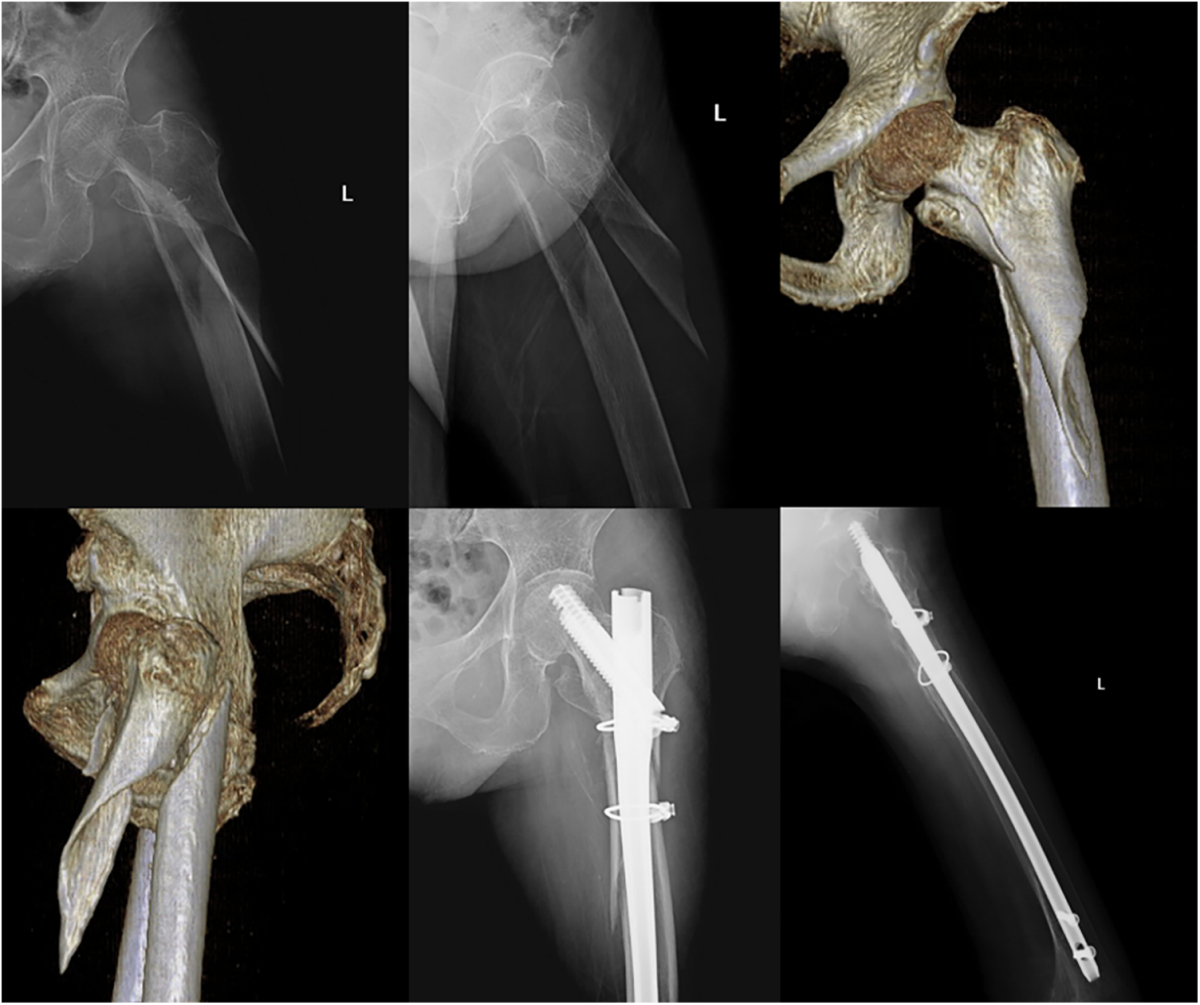
A 75-year-old patient with an unstable intertrochanteric femur fracture (AO/OTA 31 A 3.1). She received an internal fixation with InterTAN and two titanium cables since the lateral wall was relatively intact and the fracture lines involved the subtrochanteric part.

An additional incision was made anterior to the previous incision after an unsatisfied attempt by traction. A bone hook was used for temporary fixation of the lateral wall fragment. One or two titanium wires were inserted to encircle the fragment according to size (Figure 1). Wires were tightened at an approximate placement of the intramedullary nail.

### Plate group

**Figure 2:**
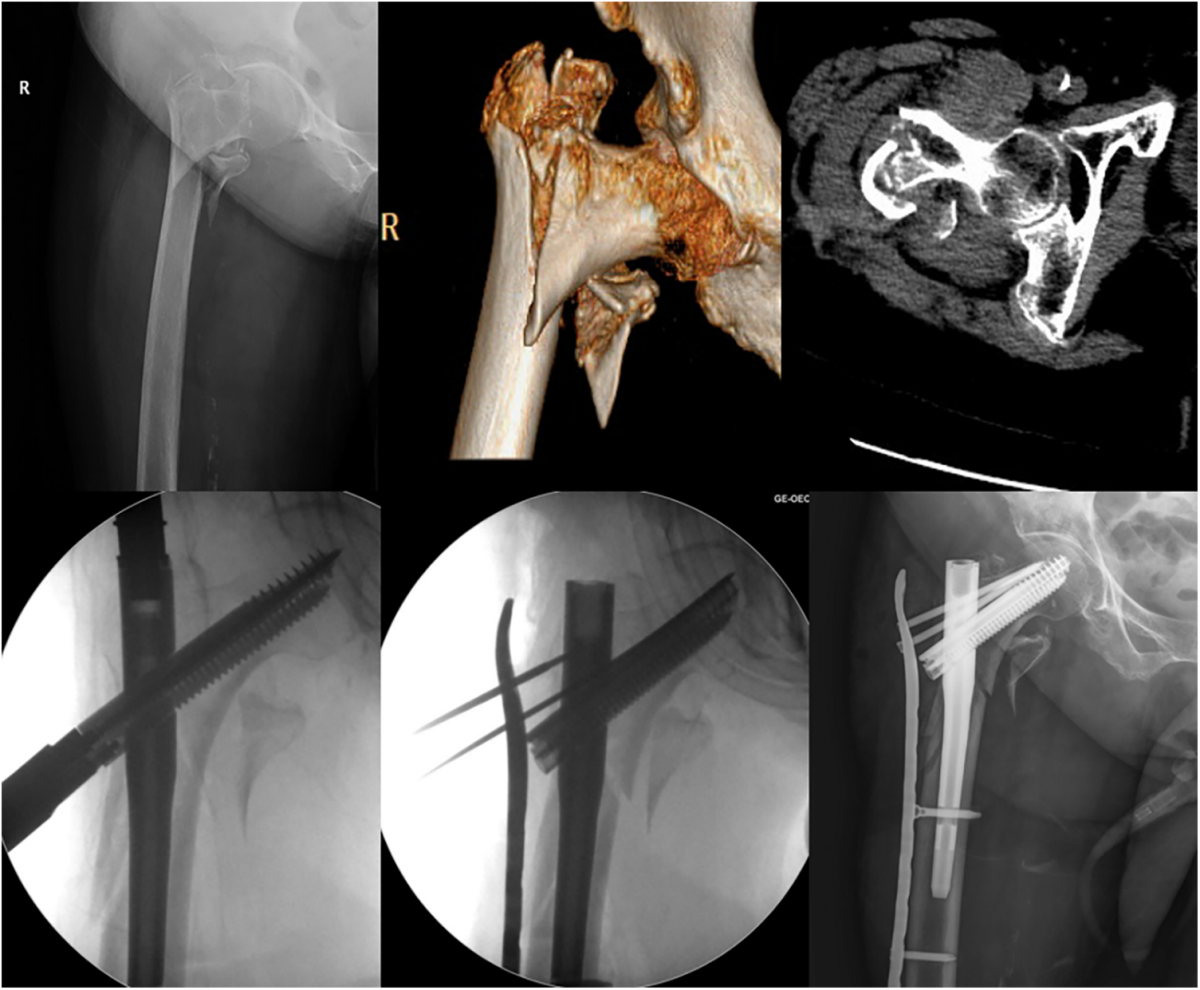
An 81-year-old female underwent an unstable intertrochanteric femur fracture (AO/OTA 31 A 2.3). She was treated with the InterTAN and a buttress plate fixation, which was used to restore the integrity of the comminuted lateral wall.

After the ideal position of the intramedullary nail was achieved, the original incision was extended distally through the apex of the greater trochanter to expose the comminuted lateral wall and periosteum surface. The plate was placed anterolateral to the lateral wall (Figure 2). Identified by fluoroscopy, several minimally invasive incisions were made to explore the locking hole and screws were placed in order.

### Perioperative treatments

A dose of 3-gram cefuroxime per day was used within three days after surgery to prevent infection (clindamycin if allergic to penicillin), and low-molecular-weight heparin sodium was performed daily within fourteen days to prevent deep venous thrombosis of lower limbs. The intravenous antibiotics continued if the infection was observed during follow-ups. According to comments from a cardiologist, perioperative blood transfusion was required for hemoglobin less than 70 g/L.

Patients were encouraged to initiate isometric contraction exercise of the quadriceps femoris and passive flexion-extension motion on the first day postoperatively. Then, they gradually began some weight-bearing activities according to their recovery with the guidance of surgeons.

### Clinical observations

Both groups' baseline data and operative details, including gender, age, injury mode, injury type, time to surgery, operative time, fluoroscopy time, blood loss and transfusion rate, were extracted from electronic records.

The patients were routinely followed up at one, two, three, six, nine, and twelve months. In our study, complications included wound condition and radiographic results, which included Z/reverse Z effect (screws backing out of the nail laterally), varus malunion (varus angulation > 10 degrees) and internal fixation breakage. Fracture union time was also recorded, referring to the absence of pain and tenderness at the hip and bridging callus in three or more cortices on anteroposterior and lateral views (11).

Hip joint function was also analyzed using the Harris Hip Scoring (HHS) system at the 1- and 3-month follow-ups. Two independent and blinded orthopedic surgeons assessed function scores and averaged their measurements. This scoring system included four aspects: pain, function, absence of deformity, and range of motion. The score standard had a maximum of 100 points. A total score <70, poor; 70–80, fair; 80–90, good; and 90–100, excellent. Meanwhile, the weight-bearing capacity was also evaluated, which was classified into three levels: full weight-bearing, partial weight-bearing (with the help of a crutch or rollator walker) and no weight-bearing (with a wheelchair).

### Statistical analysis

This study was analyzed using the statistics software SPSS (version 22.0, Chicago, IL). Descriptive statistics were performed for clinical data among two groups. Enumeration data variables were summarized as count (%), and continuous variables were mean ± standard deviation. The statistical differences for continuous variables were compared using the independent sample *t*-test. Similarly, the chi-squared test/Fisher's exact test (if any variable was less than 5) was used to assess the enumeration data. A *p*-value less than 0.05 was considered significant.

## Results

### Baseline data

**Figure 3:**
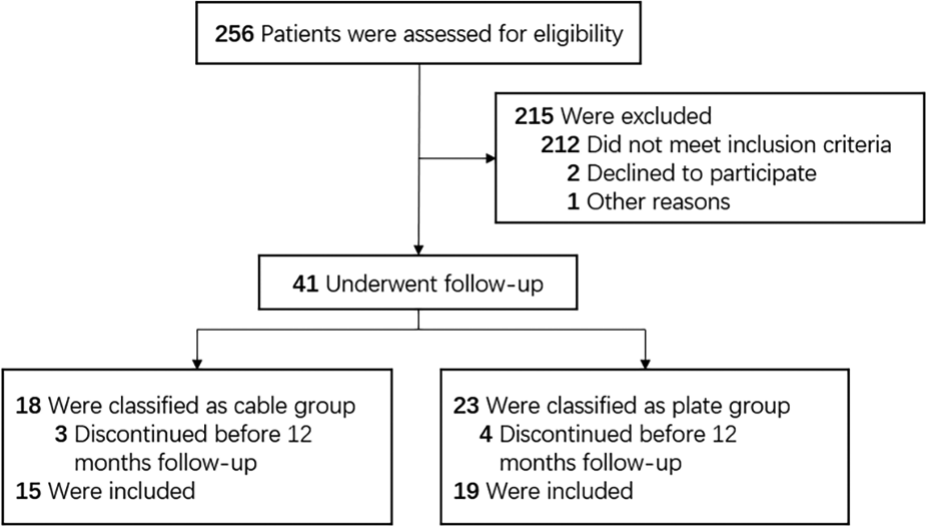
Flowchart of the study design.

Details of the patient flow are stated in Figure 3. Forty-one patients were included in this study to receive intramedullary fixations combined with buttress plates (23 patients) or cables (18 patients). The endpoint of follow-up assessments was January 2023. Among these patients, 34 (82.9%) were eligible for inclusion in this study (plate group 19 cases and cable group 15 cases).

The comparison of demographic and injury characteristics between the two groups is available in Table 1, with no significant difference. Similarly, among surgical details, the mean operative time, mean fluoroscopy time, mean length of preoperative hospitalization, mean blood loss and transfusion rate in plate group were 114.7 ± 16.3 min, 39.0 ± 7.1 times, 5.6 ± 1.3 days, 235.1 ± 46.3 ml and 15.8%, and in cable group were 103.3 ± 18.8 min, 37.7 ± 7.6 times, 5.4 ± 1.5 days, 206.3 ± 48.5 ml and 13.3% (*p* > 0.05).

**Table 1:** Characteristics of patients sustaining unstable intertrochanteric fractures.

Characteristics	Plate group (*n* = 19)	Cable group (*n* = 15)	*p* value
Gender, male, *n* (%)	10 (52.6)	7 (46.7)	0.730
Age, yrs, mean ± SD	73.1 ± 10.1	74.7 ± 9.6	0.639
Injury mode, *n* (%)			1.000
Fall	16 (84.2)	13 (86.7)	
Traffic accident	3 (15.8)	2 (13.3)	
Injury type, left, *n* (%)	10 (52.6)	8 (53.3)	0.968
AO/OTA classification, *n* (%)			0.832
31A 2.2–2.3	12 (63.2)	10 (66.7)	
31A 3.1–3.3	7 (36.8)	5 (33.3)	
Time to surgery, days, mean ± SD	5.6 ± 1.3	5.4 ± 1.5	0.713
Operative time, minutes, mean ± SD	114.7 ± 16.3	103.3 ± 18.8	0.067
Fluoroscopy time, number, mean ± SD	39.0 ± 7.1	37.7 ± 7.6	0.621
Blood loss, ml, mean ± SD	235.1 ± 46.3	206.3 ± 48.5	0.085
Transfusion rate, (%)	3/19 (15.8)	2/15 (13.3)	1.000

SD, standard deviation.

### Weight-bearing capacity and HHS system (1- and 3-month postoperatively)

The weight-bearing capacity improved over time (Table 2). In a one-month postoperatively review of the plate group, twelve patients (63.2%) could bear full weight, six (31.6%) could bear partial weight, and one (5.3%) could not bear any weight. Of the cable group, eleven patients (73.3%) could bear full weight, two (11.3%) could bear partial weight and two (11.3%) could not bear any weight. At three months, most of both groups (plate group: 84.2%, cable group: 86.7%) got rid of crutches. Only 3 (15.8%) and 2 (13.3%) in the plate and cable groups could sustain the partial weight.

**Table 2:** Clinical outcomes of patients in both groups.

Outcomes	Plate group (*n* = 19)	Cable group (*n* = 15)	*p* value
Ambulation time, days, mean ± SD	4.8 ± 1.0	5.1 ± 0.7	0.453
Time of fracture union, weeks, mean ± SD	14.7 ± 2.3	14.1 ± 1.8	0.445
Postoperatively 1-month			
Weight bearing, *n* (%)			0.380
Full weight-bearing	12 (63.2)	11 (73.3)	
Restricted weight-bearing	6 (31.6)	2 (11.3)	
No weight-bearing	1 (5.3)	2 (13.3)	
HHS, points, mean ± SD	79.9 ± 4.5	80.8 ± 4.6	0.590
Postoperatively 3-month			
Weight bearing, *n* (%)			1.000
Full weight-bearing	16 (84.2)	13 (86.7)	
Restricted weight-bearing	3 (15.8)	2 (13.3)	
No weight-bearing	0	0	
HHS, points, mean ± SD	83.6 ± 4.9	83.8 ± 3.7	0.913
Complication within 1 year, *n*			
Local wound			
Infection	0	0	/
Incision fat liquefaction	2	1	1.000
Radiographic observations			
Z/Reverse Z effect	0	0	/
Varus malunion	0	0	/
Internal fixation breakage	0	0	/

SD, standard deviation; HHS, harris hip score.

The HHS system demonstrated that these two groups were 79.9 ± 4.5 and 80.8 ± 4.6 in the first month after surgery and were 83.6 ± 4.9 and 83.8 ± 3.7 in the third month.

### Complications

The mean ambulation time and fracture union of the plate group were 4.8 ± 1.0 days and 14.7 ± 2.3 weeks; Of the cable group were 5.1 ± 0.7 days and 14.1 ± 1.8 weeks. During one year of follow-up, fat liquefaction from the surgical incision was observed in three cases (plate group: 2, cable group: 1). Finally, their wound entirely healed after a routine dressing change. In addition, neither group had abnormal radiographic signs, including Z effect, varus malunion, internal fixation breakage, and bone nonunion.

## Discussion

The lateral femoral wall has been recognized as a key predictor of reduction quality in intertrochanteric fractures. In a biomechanical study, authors found that the failure load of the broken medial wall was significantly lower than that in the lateral wall, which demonstrated that the integrity of the medial wall was the first notice to offer the bone strut (13). The intact posteromedial segment of the femur provided stability to resist the lateral wall defect. Conversely, if the posteromedial section was unstable, the bone mass of the lateral wall was decisive in intraoperative reduction quality. For all this, there was no consensus regarding the boundary of the lateral wall. In 2004, Gotfried defined it as the proximal extension of the femoral shaft (2). Palm et al. explained the lateral wall as the lateral femoral cortex distal to the vastus ridge (3). Recent literature thought the lateral wall was the lateral region between two lines, one as a tangent to the superior of the femoral neck and the other as a tangent to the inferior (14).

Over the years, an amount of fixation devices were used for unstable intertrochanteric fracture: proximal femoral locking compression plate, proximal femoral nails anti-rotation, InterTAN nail, dynamic hip screw, et al. Of these, the intramedullary fixation assisted with traction table showed outstanding therapeutic endpoints, with advantages of less trauma, less bleeding, shorter bone union time and earlier rehabilitation (15). Hsu et al. first used a quantitative index to describe the reliability of the lateral wall thickness (16). Subsequent studies verified its essential role in predicting intraoperative lateral wall fractures and suggested intramedullary nail fixation, especially when lateral wall thickness was less than 21 mm (17, 18).

However, the intramedullary fixation system cannot reconstruct the cortical support of the lateral wall. Some authors considered that the broken lateral wall should be concurrently treated to minimize the risk of postoperative fracture displacement or internal fixation failure (19). So far, the primary options for the lateral wall reconstruction include (i) the cerclage wire or titanium cable, which represents minimally invasive and additional support to the posteromedial and posterolateral fragments to achieve a better reduction. However, this method is restricted to the integrity of the lateral wall (20); (ii) the additional plate, which offers sufficient rotation stability and avoids the secondary displacement. However, it has several disadvantages, including excessive operating time and blood loss (4); (iii) reverse distal femoral locking compression plates. Some studies reported that this additional fixation failed to maintain the lateral wall in fractures with obvious subtrochanteric extension (6, 14).

A previous study compared different augmented methods for intramedullary fixation and showed that the cable group's operative time was relatively short. But the locking plate was more beneficial in clinical outcomes, including intraoperative reduction quality, time to full weight-bearing, bone healing, and function recovery (9). Jain et al. introduced a “mobile window” technique to acquire early union, lesser complication and better clinical outcomes: the auxiliary plate was slid over the guide pins till the bone after an additional small incision was made and several screws were then inserted, passing through the plate and nail (11). However, these authors observed that this method could cause significantly more surgical time, blood loss, and fluoroscopy exposure.

Our study reviewed the clinical outcomes of patients with unstable intertrochanteric fractures and treated with different augmented devices. Statistical difference in baseline data, surgical details and clinical outcome was not found between the plate and cable group (*p* > 0.05). According to the lateral wall condition, the cable or buttress plate strengthened the simple or comminuted segment (20, 21). Selection bias for additional fixations was inevitable. Notably, patients in the plate group had relatively longer operative time (114.7 min vs. 103.3 min, *p* = 0.067) and higher blood volume loss (235.1 ml vs. 206.3 ml, *p* = 0.085) than the cable group. This marginally significant was attributed to longer incisions to achieve the proper location for plates, which was similar to Wang et al. (10) Inconsistent with previous studies, we did not find any radiology-related complications (4, 10, 20). Surprisingly, no case was confined to a wheelchair at the three-month follow-up.

However, there were certain limitations in our study. Firstly, this was a non-randomized control study. The buttress plate or cable was used according to the condition of the lateral wall fragment. Secondly, our study provided operative experience and clinical results, which needed to be biomechanically verified. Hence, a relevant study should be carried out in the future. Thirdly, these operations were accomplished by a single group in our institution. Therefore, it seems unlikely to exclude surgeon bias from these results.

## Conclusions

Additional titanium cable or buttress plate is available in intramedullary fixation of unstable intertrochanteric femoral fractures. This advanced technique is beneficial to create the mechanical support and reobtain the lateral wall anatomy. Relatively satisfactory clinical outcomes could be expected after these interventions.

## Data Availability

The raw data supporting the conclusions of this article will be made available by the authors, without undue reservation.
